# Supplementary material for: Pre-Diagnosis Diet Predicts Response to Exclusive Enteral Nutrition and Correlates with Microbiome in Pediatric Crohn Disease
Source: Nutrients. 2024 Apr 2;16(7):1033. doi: 10.3390/nu16071033 (PMC11013084; doi:10.3390/nu16071033)
Supplement: Supplementary file 1 [file nutrients-16-01033-s001.zip › nutrients-2932483-supplementary.pdf]

SUPPLEMENTAL METHODS:

FFQ Kilocalorie Adjustments

The FFQ semi-quantitatively assessed dietary intake over the previous 12 months with 82 questions by providing sample portion sizes and allowing patients to select if their portions were larger, smaller, or approximately equal to the given example portion size. To provide an updated reflection of caloric values, and as the original FFQ analysis software was no longer available and current for use, the Canadian Nutrient File (CNF) online database (Health Canada, 2015. https://food-nutrition.canada.ca/) was used to generate an estimated caloric value for each FFQ item, obtained by averaging three similar items from the CNF, guided and supported by two clinical pediatric dietitians (MC & JW). Calories for items were adjusted to include modifications such as frying, use of extra-lean meats, or low-calorie and reduced-fat options. Kilocalories were adjusted based on the reported servings sizes; 50% of the calories were assigned for selections of "less than" the given serving size, and 150% of the calories were assigned for selections of "greater than". Estimated daily calories were assessed for unusual values and outliers were tested by converting values into Z-scores. No unrealistic for age and body weight values and no dietary pattern outliers were identified (no Z-scores greater than 3 or less than -3). Responses were collapsed into 43 food and beverage groups, based on nutrient similarity and common patterns of consumption with the assistance of registered dietitians (MC & JW) (**Table S3**). Food group servings per day were kilocalorie-adjusted using the Willett residuals method, as described by Willett *et al.*[1] using Stata 14 [2].

Table S1 *Centre of data collections for EEN-FFQ cohort.*

CIDsCaNN Site	EEN-FFQ Participants
Calgary – Alberta Children's Hospital	6
Edmonton – Stollery Children's Hospital	16
Halifax – Halifax Children's Hospital	24
Hamilton – McMaster Children's Hospital	13
Montreal – Montreal Children's Hospital	1
Ottawa – The Children's Hospital of Eastern Ontario	7
Ste Justine – Centre Hospitalier Universitaire Sainte-Justine	2
Toronto – The Hospital for Sick Children	25
Vancouver – BC Children's Hospital	6
Winnipeg – The Children's Hospital of Winnipeg	3

Table S2 *Stool specimen collection numbers for baseline, 6-month, 12-month, & 18-month timepoints.*

Timepoint	Stool Samples Collected
Baseline	36
6 Months	24
Baseline & 6 Month Pairs	18
12 Months	16
18 Months	14

Table S3 *FFQ Food Groups used for multivariate analysis.*

Food Group Name	FFQ Items Included in Food Group
**High Fiber Cereals**	high fiber cereals (*e.g.,* All Bran, 100% Bran, Bran flakes…)
**Other Cereals**	other breakfast cereals/hot cereals
**Sugary Condiments**	sugar added to cereals; jam, honey, sweet spreads; sugar in coffee/tea
**Refined Grains**	commercial sliced white bread, other white breads
**Whole Grains**	commercial sliced whole wheat breads, other whole wheat breads
**Rice etc.**	rice, rice noodles, couscous
**Salty Snacks**	salty snacks (chips, crackers, pretzels, popcorn)
**Nuts/Seeds**	peanut butter; sunflower seeds; nuts, peanuts, other seeds
**Vegetable Soup**	tomato or vegetable soups
**Other**	other soups
**High fat Potato**	French fries or pan-fried potatoes

**Potato**	boiled, mashed, or baked potato
**Margarine**	margarine
**Butter**	butter, on bread or on cooked vegetables
**Red Meat**	beef, other Meats (veal, lamb, game…)
**Pork**	pork
**Liver/Organ Meats**	liver, other organ meats
**White Meat**	chicken, turkey
**Processed Meat**	ham, cold cuts, sausages hot dogs
**Meat Sauce**	sauces (brown, white, BBQ, gravy…)
**Fish**	salmon, trout, sardines, herring, tuna, other fish (sole, cod, fish sticks…)
**Seafood**	seafood
**Pasta**	pasta with tomato sauce with or without meat, Pasta with creamy sauce
**Pizza**	pizza
**Legumes & Pulses (Excluding soy)**	beans, peas, lentils, hummus
**Vegetable Protein Soy**	tofu, soya
**Eggs**	eggs
**Vegetables**	green/yellow beans, green peas, corn; carrots; tomatoes; green, red, yellow sweet peppers; other vegetables; leafy greens; cruciferous vegetables
**Vegetable Juice**	tomato or vegetable juices
**Salad dressings, mayonnaise, dips**	salad dressings, mayonnaise, dips
**Cheese**	cheese
**Yogurt**	yogurt
**Fruit**	apples, pears; bananas; melons; oranges, grapefruits, tangerines; other fruits, berries
**Sugary Desserts**	cakes, pies, doughnuts, pastries; muffins; cookies; candies, chocolate; milk-based desserts; ice cream
**Pure Fruit Juice**	pure fruit juice – 100%
**Sugar Sweetened Beverages**	fruit drinks (sugar added), soda
**Diet Soda**	diet soft drinks
**Full Fat Milk**	whole milk, milk or cream in coffee/tea
**Reduced Fat Milk**	1%, 2% or skim milk

**Milk Alternatives**	soya milk
**Coffee**	coffee
**Alcohol**	beer; table wine, aperitifs; spirits
**Granola bars, chewy bars, cereal bars**	granola bars, chewy bars, cereal bars

***Table S4** PC Eigenvalues and % of dietary variance explained.*

**Component**	**Eigenvalue**	**% Of Variance**
Principal Component 1	3.41	7.4
Principal Component 2	3.38	7.35
Principal Component 3	3.29	7.16
Principal Component 4	3.23	7.01
Principal Component 5	2.36	5.13
Principal Component 6	2.17	4.71
Principal Component 7	1.96	4.27
Principal Component 8	1.91	4.15
Principal Component 9	1.78	3.87
Principal Component 10	1.52	3.29

Relative Mediterranean Diet

To assess for associations with a MD, we used a tertile defined scoring system to calculate the relative Mediterranean Diet (rMED). The 82 food items assessed in the FFQ were assigned to one of the previously listed categories when possible and used to create the rMED scoring index summarized in materials and methods. Briefly, based on Buckland et al (2009), A value of 1, 2, or 3 was assigned to the first, second, and third tertiles of intake, respectively, positively scoring higher intakes for the 6 components presumed to fit the Mediterranean diet: fruit, vegetables, legumes, cereals, fresh fish, and olive oil and lower scores for total meat and dairy products. No red wine intake was reported, so this food group was not utilized. A range of scores from 10 to 21 was obtained (full range: 8-24).

Microbiome Analysis

Approximately 0.2-0.3g of sample was added to 800µL of 200 mM $NaPO_4$ (pH 8), and 100µL of guanidine thiocyanate-ethylenediaminetetraacetic acid (EDTA)–Sarkosyl along with 0.2g of 0.1mm glass beads and 0.2g of 2.8mm ceramic beads (Mo Bio, Carlsbad, CA). Mechanical lysis was carried out in a Powerlyzer (Mo-Bio) at 3,000 RPMs for three minutes. This was followed by two steps of enzymatic treatment. First, 50µL lysozyme (100 mg mL-1) and 10µL RNase A (10 mg mL-1) were added and incubated at 37°C for 60 minutes. Second, 25µL 25% sodium dodecyl sulfate (SDS), 62.5µL 5 M NaCl, and 25µL proteinase K were added to the sample and incubated at 65°C for 30 minutes. Samples were pelleted at 13,500 × g for five minutes by centrifugation and the supernatant was recovered. Genomic DNA was purified using automated DNA extraction on a MagMAX (Thermo Fisher Scientific) as per manufacturer's instructions.

The 16S rRNA variable region 3 through variable region 4 was amplified in triplicate using 341F and 806R 16S rRNA primers modified for the Illumina platform with adaptors containing 6-base pair unique barcodes to the reverse primer as described previously [3]. Each sample reaction mixture contained 5pmol of each primer, 200µmol L-1 of each deoxynucleoside triphosphate (dNTP), 1.5 mM $MgCl_2$, and 1U Taq polymerase (Life Technologies, Carlsbad). The PCR protocol consisted of an initial denaturation step at 95°C for five minutes, 30 cycles, each step for 30 seconds, of 95°C, 50°C, and 72°C, and a final extension step at 72°C for seven minutes. Triplicate reactions were pooled, and the amplicons were then sequenced (2x300 paired end) using the Illumina MiSeq platform.

Paired-end reads were merged, demultiplexed and conducted quality control implementation using QIIME2 [4] pipeline with DADA2 [5], which we refer to herein as amplicon sequences variants (ASV). An even depth of 8,938 sequences per sample was used to conduct microbiome diversity. We assigned the sequences to taxonomic categories including kingdom, phylum, class, order, family and genus levels using a pre-trained Naive Bayes classifier based on Silva 132 99% ASVs database [6]. The raw sequencing data is deposited into the Sequence Read Archive (SRA) of NCBI (http://www.ncbi.nlm.nih.gov/sra) under BioProject PRJNAXXXX.

SUPPLEMENTRY FIGURE LEGENDS

Figure S1 Diet Pattern Associations with rMED Scores.

Vegetarian and Mature dietary adherence were positively associated with rMED score (rho=0.2035, p<0.05; rho=0.5989, p<0.0001, respectively), Pre-packaged diet adherence had a trending positive association with rMED score (rho=0.1907, p=0.0537). Meat diet adherence was not significantly associated with rMED score.

Figure S2 Mature diet adherence is positively associated with age.

rho=0.3586, p<0.001

Figure S3 food group intakes between Meat tertiles in males, and Mature tertiles in all patients.

A: Comparison of kCal adjusted food group daily servings by Meat diet adherence tertile in males. **B**: Comparison of kCal adjusted food group daily servings by Mature diet adherence tertile. **C**: Comparison of kCal adjusted food group daily servings by rMED tertile. Tertile 1 represents lowest adherence, 3 the highest. # = p 0.050033, **** = p<0.0001, ** = p<0.01, *=p<0.05.

Commented [M1]: No c in Figure S3, please revise.

Figure S4 Food group intakes between Pre-packaged tertiles in males, and rMED tertiles in all patients.

A: Comparison of kCal adjusted food group daily servings by Pre-packaged diet adherence tertile in males. **B**: Comparison of kCal adjusted food group daily servings by rMED tertile. Tertile 1 represents lowest adherence, 3 the highest. **** = p<0.0001, ** = p<0.01, *=p<0.05.

Figure S5 Sex-specific diet differences and associations between diet adherence and disease location.

A-B: Males had significantly higher adherence to a mature diet (p<0.05) and trending higher rMED scores. **C-D**: Females with Paris L are more adherent to a Mature diet and less adherent to a Pre-packaged diet than females with Paris L2/L3 **=p<0.01.

Figure S6 Sex-Specific Associations with PGA

A: At baseline females had a significantly higher PGA compared to males (p<0.05). **B**: females saw a significantly greater drop in PGA from baseline (p<0.01). *=p<0.05, **=p<0.01

SUPPLEMENTRY FIGURES

Figure S1 Diet Pattern Associations with rMED Scores.

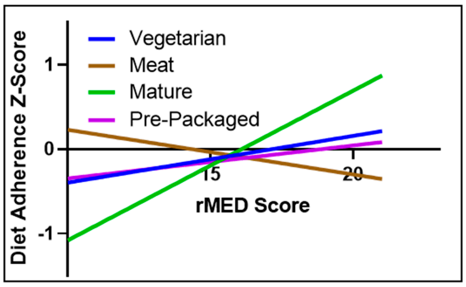

Figure S2 Mature diet adherence is positively associated with age.

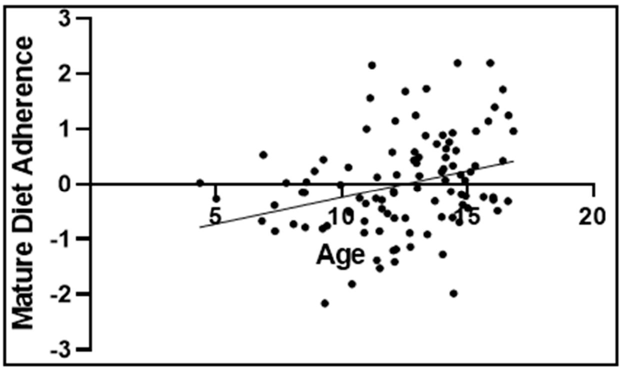

Figure S3 food group intakes for Meat tertiles in males, and Mature tertiles in all patients.

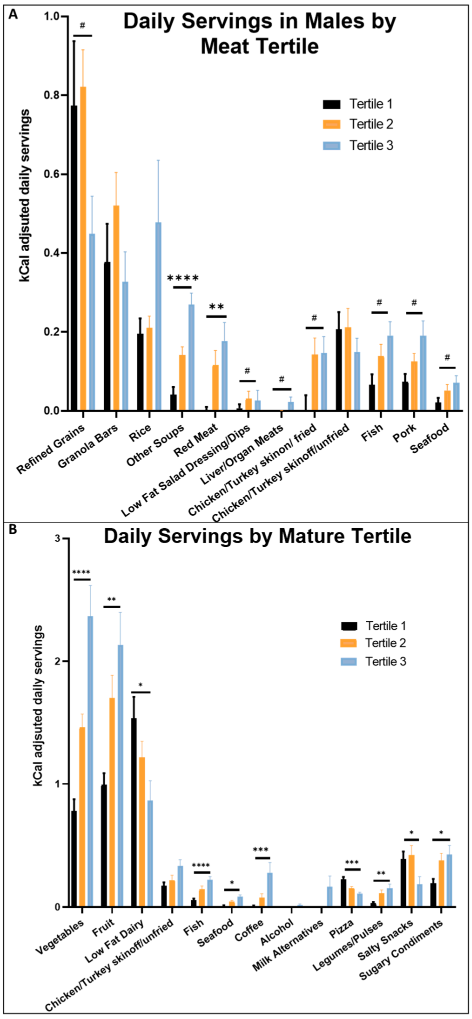

Figure S4 Food group intakes for Pre-packaged tertiles in males, and rMED tertiles in all patients.

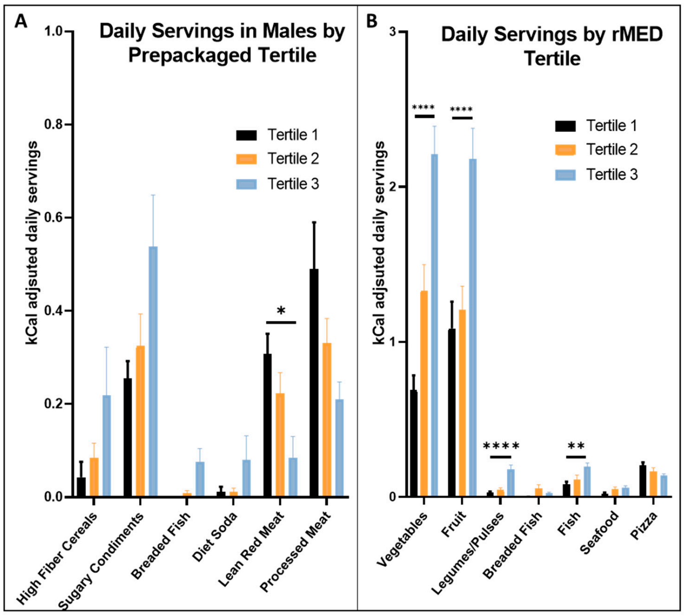
A
Daily Servings in Males by Prepackaged Tertile
kCal adjsuted daily servings
1.0
0.8
0.6
0.4
0.2
0.0
Tertile 1
Tertile 2
Tertile 3
*
High Fiber Cereals
Sugary Condiments
Breaded Fish
Diet Soda
Lean Red Meat
Processed Meat
B
Daily Servings by rMED Tertile
kCal adjsuted daily servings
3
2
1
0
****
****
****
**
Vegetables
Fruit
Legumes/Pulses
Breaded Fish
Fish
Seafood
Pizza

Figure S5 Sex-specific diet differences and associations between diet adherence and disease location.

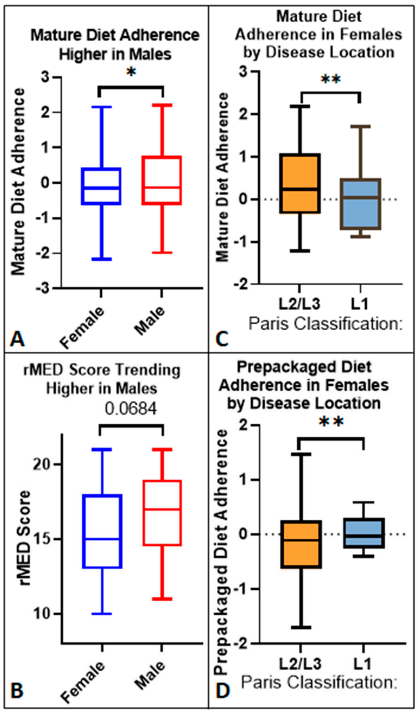

Figure S6 Sex-Specific Associations with PGA

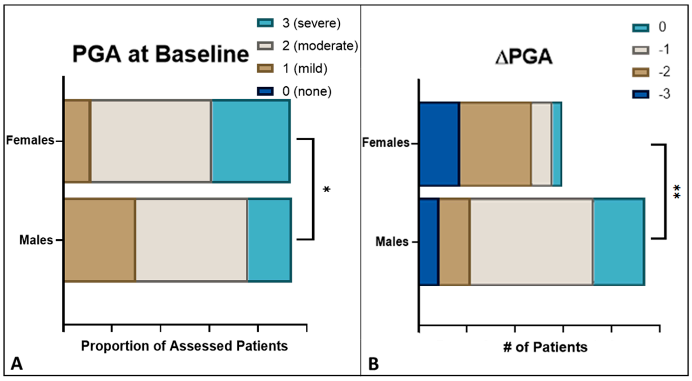

Supplementary References:

1. Willett, W.C.; Howe, G.R.; Kushi, L.H. Adjustment for total energy intake in epidemiologic studies. *The American journal of clinical nutrition* **1997**, *65*, 1220S-1228S.
2. StataCorp. Stata Statistical Software: Release 14. **2015**.
3. Szamosi, J.C.; Forbes, J.D.; Copeland, J.K.; Knox, N.C.; Shekarriz, S.; Rossi, L.; Graham, M.; Bonner, C.; Guttman, D.S.; Van Domselaar, G. Assessment of inter-laboratory variation in the characterization and analysis of the mucosal microbiota in Crohn's disease and ulcerative colitis. *Frontiers in Microbiology* **2020**, *11*, 2028.
4. Bolyen, E.; Rideout, J.R.; Dillon, M.R.; Bokulich, N.A.; Abnet, C.C.; Al-Ghalith, G.A.; Alexander, H.; Alm, E.J.; Arumugam, M.; Asnicar, F.; et al. Reproducible, interactive, scalable and extensible microbiome data science using QIIME 2. *Nat Biotechnol* **2019**, *37*, 852-857, doi:10.1038/s41587-019-0209-9.
5. Callahan, B.J.; McMurdie, P.J.; Rosen, M.J.; Han, A.W.; Johnson, A.J.; Holmes, S.P. DADA2: High-resolution sample inference from Illumina amplicon data. *Nat Methods* **2016**, *13*, 581-583, doi:10.1038/nmeth.3869.
6. Wang, Q.; Garrity, G.M.; Tiedje, J.M.; Cole, J.R. Naive Bayesian classifier for rapid assignment of rRNA sequences into the new bacterial taxonomy. *Appl Environ Microbiol* **2007**, *73*, 5261-5267, doi:10.1128/AEM.00062-07.
